# Will We Fly Again? Modeling Air Travel Demand in light of COVID-19 through a London Case Study

**DOI:** 10.1177/03611981211025287

**Published:** 2021-07-14

**Authors:** Francesco Manca, Aruna Sivakumar, Jacek Pawlak, Norbert J Brodzinski

**Affiliations:** 1 Urban Systems Lab, Centre for Transport Studies, Department of Civil and Environmental Engineering, Imperial College London, London, U.K

## Abstract

The COVID-19 pandemic and associated travel restrictions have created an unprecedented challenge for the air transport industry, which before the pandemic was facing almost the exact opposite set of problems. Instead of the growing demand and need for capacity expansion warring against environmental concerns, the sector is now facing a slump in demand and the continuing uncertainty about the impacts of the pandemic on people’s willingness to fly. To shed light on consumer attitudes toward air travel during and post the pandemic, this study presents an analysis that draws on recently collected survey data (April–July 2020), including both revealed and stated preference components, of 388 respondents who traveled from one of the six London, U.K., airports in 2019. Several travel scenarios considering the circumstances and attitudes related to COVID-19 are explored. The data is analyzed using a hybrid choice model to integrate latent constructs related to attitudinal characteristics. The analysis confirms the impact of consumers’ health concerns on their willingness to travel, as a function of travel characteristics, that is, cost and number of transfers. It also provides insights into preference heterogeneity as a function of sociodemographic characteristics. However, no significant effects are observed concerning perceptions of safety arising from wearing a mask, or concerns over the necessity to quarantine. Results also suggest that some respondents may perceive virtual substitutes for business travel, for example video calls and similar software, as only a temporary measure, and seek to return to traveling as soon as it is possible to do so safely.

The ongoing COVID-19 pandemic has affected air travel to an unprecedented extent, leading to the worst-ever crisis of the air transport sector (*1*). Airlines worldwide have faced a huge drop in demand, for example 98% drop in passengers for 6 weeks in a row over April and May 2020, as stated by the Airport Council International Europe (*2*). Airlines and airports face the challenging task of dealing with the constantly changing policies of governments, often lacking coordination both at the national and international levels. In this context, finding the right balance between breaking even and taking the necessary, though costly, measures to guarantee the safety of travelers is no trivial task. These measures can include social distancing at the airports and on-board the airplanes (e.g., empty middle seat, boarding by row number), providing sanitizing gels, masks and gloves, and conducting body temperature checks, or even COVID-19 tests, before departure and/or after landing. Under these new circumstances, not only is the travel experience likely to change but also the air travel itinerary might evolve in terms of the cost of the ticket and the time required at the airport before departure and after arrival.

Before the pandemic, the air travel sector was experiencing sustained growth, expected to continue at the rate of 3.5% per year to reach 8.2 bn air travelers by 2037 (*3*). However, this ongoing growth has been also looked on with increasing environmental awareness and concern because of the associated carbon emissions, at present around 2% of all global carbon emissions (*4*). A particularly visible manifestation of this growing environmental concern was the emergence and spread, initially in Sweden in 2017 but subsequently globally, of the concept of “flight shaming”, derived from the Swedish expression “flygskam”. The concept attracted mainstream attention in the media and its effect was expected to continue, translating into a higher willingness to replace air travel with other, more sustainable alternatives, especially rail, and change of habits, for example reduction in long-distance travel, local tourism, or replacement of trips with virtual alternatives, such as videoconferencing (*5*). Unsurprisingly, therefore, airlines in 2019 were strongly oriented toward dealing with their environmental impacts, for example through expanding their carbon offsetting programs (*6*).

In this environment, the rapid and unpredicted onset and scale of the COVID-19 pandemic brought a major shock to the air industry, shifting the attention toward means of survival in a post-pandemic world. The introduction of necessary measures to ensure the safety of travelers is being accompanied by adjustments in operations, for example prompting fleet reductions and early fleet retirements (e.g., Boeing 747 by British Airways and Qantas) or staff reductions (*7*). And yet, substantial uncertainty persists in the understanding of how air passenger preferences might have evolved as a result of the pandemic, and which measures implemented by the air industry and governments could prove the most effective in dealing with the medium-to-long term impacts of the pandemic on air travel demand.

It is the objective of this paper to provide some insight into these issues, drawing on recently collected online survey data from London, U.K. The dataset is unique, as it comprises information from a revealed preference (RP) survey concerning the most recent air trip made by the respondent before January 2020, that is, before the restrictions caused by the pandemic, as well as from a stated preference (SP) survey which explored several hypothetical travel scenarios, including a specific SP exercise that took into account scenarios and attitudes related to COVID-19. In this paper, the data related to COVID-19 is analyzed using the hybrid choice modeling (HCM) approach, which makes it possible to integrate latent constructs, for example based on psychometric indicators, into the discrete choice models of air travel decisions. The paper provides a novel set of insights into how people make air travel-related decisions in the context of the pandemic, including trade-offs between cost and time, while taking into account safety perceptions and attitudes related to the pandemic.

The rest of this paper is structured as follows. The next section briefly presents an overview of the challenges faced by the air transport sector before COVID-19 and summarizes the current literature on modeling air travel demand. The section after that presents the data and the methodology adopted in this research. The penultimate section presents and discusses the substantive results, and the final section concludes the paper.

## Literature Review

Air travel provides a vital means of transport which has so far been crucial for the functioning of global economies, including facilitation of business links and enabling tourism, but also for maintaining social cohesiveness, for example in geographically vast countries. It facilitates trade and contributes to enhanced productivity by attracting investors, supporting innovation, and improving business efficiency. Before the COVID-19 pandemic, the air transport industry had been steadily growing and this growth rate was expected to exceed the planned capacity increases and lead to an increasingly negative environmental impact. Accordingly, air travel demand modeling research efforts in the pre-COVID era were mainly focused on: (*a*) understanding and modeling environmental impacts of aviation; (*b*) demand forecasting and management, particularly in relation to dealing with “excess demand”, but also for the purpose of fleet and route planning, and in relation to regional economic and social impacts (*8*–*15*).

As regards the literature on air travel behavior, there have been several studies that use discrete choice modeling techniques to investigate the air travel attributes affecting passengers’ choice of airport, airline, and itinerary. For example, using RP data, Ashford and Benchemam developed a multinomial logit model exploring the choice behavior of business and leisure passengers in the U.K., making choices among available airports rather than establishing the airport catchment areas (*16*). Ozoka and Ashford analyzed the characteristics (i.e., access time, frequency, fare) influencing passengers’ choice of airports in a developing country (Nigeria) to assist in the planning of aviation systems (*17*).

Using more advanced data collection techniques, including both RP and SP observations, Proussaloglou and Koppelman gained insights into the trade-offs made by air travelers from Chicago and Dallas, U.S., when choosing among different airlines, flights and fare classes (*18*). This study estimated travelers’ willingness to pay for different aspects of the air travel itinerary, with passengers demonstrating higher price sensitivity for leisure travel than business travel, and the strong influence of frequent-flyer programs on the choice behavior of frequent travelers. Warburg et al. modeled how demographics (e.g., gender, income, frequent-flyer program membership) and unobserved heterogeneity affect domestic U.S. air travelers’ sensitivity to the service while making itinerary-related choices (*19*). They highlighted the importance of considering: (*a*) demographic- and trip-related interactions with service characteristics (to reveal the variations across traveler and trip segments); and (*b*) random taste variations across individuals to produce more consistent estimates of the willingness-to-pay (WTP) values. Hess et al. investigated how the use of SP data (collected in the U.S.) for air travel behavior could address the issue of limited information about the non-chosen alternatives (typical with RP data collected from departing passengers), thus enabling more explicit modeling of airfares, while also capturing the heterogeneity across different population segments (*20*).

However, all the above studies were undertaken in very different conditions, that is, in a world not affected by the COVID-19 pandemic. The only possibly comparable situation was the September 11, 2001 (9/11) shock, which stimulated analysis of passengers’ concerns about the safety of air travel and the long inspection times at airports (*21*). However, neither the 9/11 shock nor the 2008 financial crisis has had an impact on the air travel sector similar to the one caused by the COVID-19 pandemic (*22*). More recent efforts undertaken by the air industry, including the International Air Transport Association (IATA), are based on aggregate analyses of global demand using indicators such as travel search and ticketing data (*23*). In today’s post-COVID-19 world, where spending time in confined spaces and in proximity to other individuals may be a factor contributing to the risk of infection, with potentially severe health implications or even death, there is a need to develop a fresh understanding of air traveler preferences. This was the motivation behind the collection of the survey data used in this study, with an SP design to contextualize air travel choice in this period of uncertainty and, therefore, consider the impact of the pandemic on individual choice behavior. To the best of the authors’ knowledge, this is the first study using discrete choice modeling techniques to account for the endogenous effect of COVID-19 in air travel demand analysis. As part of the study, it is aimed to assess the impacts of airline and airport measures for disease control (e.g., reduced capacity in the aircraft as described by Walton) on air passenger choice (*24*). The implications of this demand assessment are of vital importance to an industry that is financially severely handicapped as a result of COVD-19 (*2*).

## Methodology

This paper develops a model of air travel choice accounting for the impacts of COVID-19 that can affect passenger decisions. Four main blocks of information have been used in the empirical analysis presented in this paper: socio-economic characteristics of respondents; factors related to the COVID-19 pandemic; personality traits of the survey respondents; and SP data on air travel choices as affected by the pandemic.

### Study Context

The data for this study was collected as part of a cross-national survey undertaken for the research project titled Airport Capacity Consequences Leveraging Aviation Integrated Modelling (ACCLAIM). An online survey was conducted in London between April and June 2020 through Panelbase (www.panelbase.net), a U.K.-based market research company specializing in providing access to online panels of respondents. The overall aim of the ACCLAIM project is to investigate a variety of factors that influence air passengers’ choice of itinerary, using data from a survey conducted in four different multi-airport regions in the world (Greater London, Shanghai, New York, and Sao Paulo). This paper focuses on analyses of the London data, as data collection for the remaining cities was ongoing at the time of writing of this paper.

The survey was administered in two waves. The first wave comprised a series of RP questions concerning the most recent air trip made by the respondent before January 2020, in addition to several questions about the respondent’s sociodemographic characteristics. Respondents who reported their most recent journey before January 2019 were excluded because of potential recall bias. During the first wave of the survey, to avoid interviewing only people traveling for personal reasons, a quota sampling technique was employed to reach a share of at least 30% and at most 40% of people traveling for business and at least 60% and at most 70% of people traveling for personal reasons. The second wave of the survey was administered as a follow-up to the pool of respondents who completed the RP wave and comprised a series of SP choice experiments. The analysis presented in this paper uses the second of two blocks of SP experiments presented to the respondents in the second wave of the survey.

The COVID-19 part of the survey for London consists of 388 respondents with complete responses, representing a broad range of trip purposes and socio-economic characteristics as shown in Table 1. The share between business and personal travelers and the distribution of income and age in the survey sample are similar to those of air travel passengers at the Greater London Area airports (*25*). In particular, according to the Civil Aviation Authority (CAA), the share of passengers on international versus domestic flights in 2018 was 94% and 6%, respectively (in the sample, 92% and 8%) and the share of travelers for business and personal reasons was, respectively, 23% and 77% (in the sample, 19% and 81%). As regards the passenger age, a marked difference can be observed between the CAA data and the sample for the age group “35 to 44” (the proportion of this age group in the sample is 14% higher than the CAA figures). However, CAA reports 6% of travelers in the age group below 18 years. These individuals could not be captured in the data collection, and are part of the reason for the differences. Finally, the income distribution in the sample is very similar to the distribution in the CAA data. The main differences are observed for the income category “£50,000 to £99,999” (CAA: 28%, sample: 36%) and for the income category “£100,000 or more” (CAA: 16%, sample: 9%).

**Table 1.** Frequency Analysis of the Sample

Variable	Classes	Percentage
Travel purpose (multiple choices were allowed)	Business	19%
	Charity and volunteering	3%
	Events	6%
	Health	3%
	Personal and social	40%
	Religious and reflective	3%
	Tourism	55%
Gender	Male	49%
	Female	51%
Age (years)	18–24	6%
	25–34	21%
	35–44	31%
	45–59	27%
	60–74	15%
	75+	1%
Income	Below £10,000	3%
	£10,000–£14,999	3%
	£15,000–£24,999	8%
	£25,000–£49,999	33%
	£50,000–£99,999	36%
	£100,000 or more	9%
	No information	9%
Primary citizenship	U.K.	89%
	EU	8%
	Non-U.K./EU	3%
Education	No schooling	0%
	Elementary or primary school	0%
	Secondary school	11%
	High school	10%
	Vocational, technical school, or equivalent	12%
	Bachelor’s degree	40%
	Master’s degree	22%
	Doctorate	4%
Employment	Working: full-time employee	62%
	Working: part-time employee	11%
	Working: self-employed	10%
	Not working: retired	10%
	Not working: student	2%
	Not working: unemployed	3%
	No information	1%
Job role	Clerical and support	18%
	Technicians or associate professionals	11%
	Managers without employee supervision responsibilities	10%
	Managers with employee supervision responsibilities	21%
	Senior managerial and directorial	9%
	Executive level	7%
	Other, or no information	24%
Number of household members	One	31%
	Two	21%
	Three	23%
	Four	17%
	Five	5%
	Six and more	2%
	No information	2%

### COVID-19 Impact, Safety Perception, and Personality Traits

In the COVID-19 parts of the survey, the respondents first replied to a series of questions about their frequency of use of video calls with family and friends living in other cities (in the U.K. or abroad) and frequency of use of online/virtual software in place of flying for business/work, before, during, and after (anticipated behavior) the pandemic. Subsequently, the respondents were presented with various statements about the coming months when, hypothetically, travel and other restrictions are completely lifted. The statements explored the safety concerns of the respondents when traveling again, and asked for their level of agreement on a 5-point Likert scale ranging from strongly disagree to strongly agree. These statements relate to the following aspects:

- being afraid of catching COVID-19
- passing COVID-19 to family and friends
- catching the virus on the airplane
- preferring not to travel to avoid catching COVID-19
- feeling safe wearing masks
- not willing to quarantine on arrival or return.

The last block of questions in the survey included statements to investigate the “Big Five” personality traits of the respondents, that is, extraversion (“outgoing, sociable” or the reverse “reserved”), agreeableness (“generally trusting” or “tends to find fault with others”), conscientiousness (“does a thorough job” or “tends to be lazy”), neuroticism (“relaxed, handles stress well” or “gets nervous easily”), openness (“active imagination” or “few artistic interests”) (*26*–*28*). This block of questions was also evaluated on a 5-point Likert scale ranging from strongly disagree to strongly agree, and was presented before the SP choice experiments.

### SP Experimental Design

SP experiments are extensively used in transport research to investigate the independent effect of attributes on SPs of the individual given a range of realistic, hypothetical scenarios (*29*). The selection of different attributes and their levels in the experiment makes it possible to identify the statistical relationships in the data and to understand the trade-offs made by the individual during the choice process (*29*).

This approach is conceptually similar to that of Holguín-Veras et al., who looked at inspection time at airports to increase passenger safety following the 9/11 attacks (*21*). In the SP survey, respondents were asked to think about the circumstances of a hypothetical air travel trip (for the same purpose as the travel reported in the RP wave). This hypothetical trip was supposed to take place when travel restrictions are lifted but there is potentially still a risk of infection. The respondents were asked to consider that the airports and the airlines would be taking measures to guarantee the safety of the travelers, including social distancing (e.g., empty middle seat), providing masks and gloves, and providing COVID-19 tests before departing and/or after landing. These could affect the attributes of the alternatives: the fare in £ (round trip per person), the total time at the departure airport, the total time at the arrival airport, and the number of transfers (0 or 1+). People could choose between two possible travel options and a no-travel option (“prefer not to travel”). The two possible travel options are characterized by the levels of the attributes which depend on the type of flight: short-haul (assuming a reference cost of £80), medium-haul (assuming a reference cost of £250) and long-haul (assuming a reference cost of £500) as shown in Table 2.

**Table 2.** Attributes Levels of Stated Preference (SP) Experiment

	Attribute levels
Segment	Short-haul
Fare (£) (round trip per person)	80/160/240
Total time (h) at the departure airport	2/4/6
Total time (h) at the arrival airport	2/4/6
Transfer	Yes/No
Segment	Medium-haul
Fare (£) (round trip per person)	250/450/650
Total time (h) at the departure airport	2/4/6
Total time (h) at the arrival airport	2/4/6
Transfer	Yes/No
Segment	Long-haul
Fare in (£) (round trip per person)	500/800/1,100
Total time (h) at the departure airport	2/4/6
Total time (h) at the arrival airport	2/4/6
Transfer	Yes/No

Following the example presented in Bliemer and Rose, an efficient SP survey design was generated with the help of the Ngene software (*30*, *31*). This type of design minimizes the standard error for the parameter estimates to obtain statistically significant model results. A heterogeneous pivot design with equal segment weights (i.e., 0.33 for the three possible short-, medium-, and long-haul segments) to calculate the Fisher Information Matrix was used to tailor the scenarios to each respondent (*32*). However, it was decided not to keep the pivoting alternative as is done in the classical pivot design, but instead to let the level vary. This is preferred because the pivoting alternative (i.e., pre-pandemic) would have always been dominant with respect to the alternatives affected by COVID-19, in which costs and times are always assumed to be greater than or at best equal to the pre-pandemic numbers. The scenarios were then divided into three blocks so that each individual replied to six choice scenarios with two of each haul segment. Moreover, given that the pandemic circumstances present an unprecedented situation, and, therefore, not having previous information on priors, it was decided to assume prior parameter values of zero. For this reason, the utility balance among the different alternatives was checked through a Monte Carlo simulation using a dataset created with the results of the efficient SP design.

The final sample used for the choice model estimations includes 2,316 observations from the sample of 388 respondents. At first glance, among these 2,316 observations, 60% of the choices were for either of the two travel options, and 40% for the no-travel option. Among the 60% of travel choices, 76% were hypothetical trips for personal purposes, and 24% trips for business; also among the 60% of travel choices, 36% referred to short-haul flights, 32% to medium-haul flights, and 32% to long-haul flights. As regards the 40% of no-travel choices, 89% were hypothetical trips for personal purposes, and 11% trips for business; whereas 29% referred to short-haul flights, 34% to medium-haul flights, and 36% to long-haul flights. To shed light on the effects of the design attributes on the respondents’ choices, a discrete choice modeling approach was employed.

**Figure 1.**
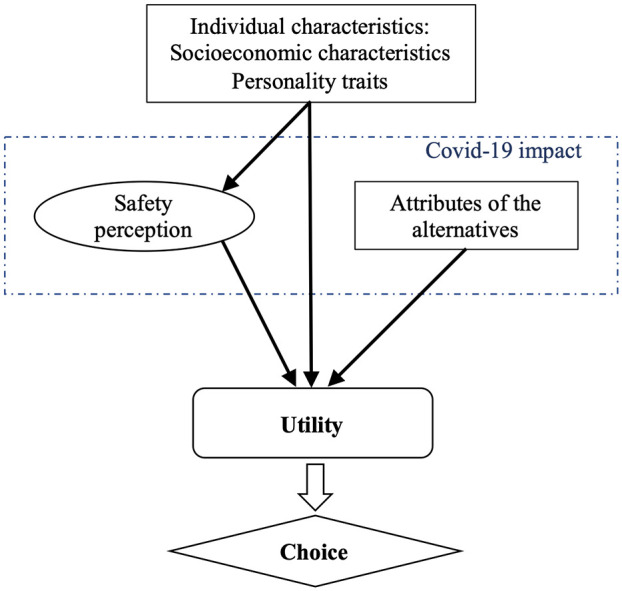
Impact of COVID-19 on the decision-making process

Throughout this study, the working assumption is that the decision-making process can be affected by the various aspects characterizing the flights available during the pandemic period. As indicated previously, measures and restrictions that need to be enforced are going to affect cost and/or time at the airports on the one hand, but also the pandemic is likely to affect the perception of safety for the traveler. Therefore, it is argued that the choice can be affected as shown in Figure 1 below:

### Modeling Methodology

Exploring the effect of the different types of factors influencing the decision-making process of the individual as presented in Figure 1, an HCM approach has been employed. HCMs provide the ability to include psychometric and other unobservable measures (i.e., safety perception) within a discrete choice model formulation which includes the attributes of the alternatives (*33*). As explained by Vij and Walker, the HCM framework has the benefit of incorporating structural relationships between observable and latent variables, which enables correcting for measurement errors and reduces the variance of the estimates (*34*). Therefore, the approach addresses some of the criticisms of standard discrete choice models and more effectively incorporates considerations highlighted in behavioral economics. Besides, HCMs can better support practice and policy as they offer greater insight into the heterogeneity in individual choice. Following Walker and Ben-Akiva et al., the mathematical specification of the HCM has three components (*35*, *36*). The first is the choice model component which shows the utility of the individual i , $U_{\mathrm{jit}}$ , associated with the alternative j in the choice task t=[1,…,T] :

(1) $$U_{\mathrm{jit}}=\mathrm{AS}C_{j}+\beta_{\mathrm{jX}}X_{\mathrm{jit}}+\beta_{\mathrm{jS}}S_{i}+\beta_{\mathrm{jA}}A_{i}+\eta_{\mathrm{ji}}+\varepsilon_{\mathrm{jit}}$$

where:

$\mathrm{AS}C_{j}$ is the alternative-specific constant;

$X_{\mathrm{jit}}$ represents the attributes of the alternatives in the choice task *t*;

$S_{i}$ represents the socio-economic characteristics of the respondents;

$A_{i}$ includes the possible latent variables;

$\beta_{\mathrm{jX}}$ , $\beta_{\mathrm{jS}}$ , and $\beta_{\mathrm{jA}}$ are the parameters to be estimated; and

$\varepsilon_{\mathrm{jit}}$ is the error term characterizing the logit model, which is assumed to be identically and independently distributed extreme value type 1 (EV1).

To account for the panel effect among the responses of the same individual i , the error component $\eta_{\mathrm{ji}}$ is assumed to be normally distributed $N\left(0,\sigma_{\eta }\right)$ where $\sigma_{\eta }$ is the standard deviation to be estimated.

The second component of the HCM is the structural model component relating the latent variable $A_{i}$ to the socio-economic characteristics of the individual *i, S′_i_*.

(2) $$A_{i}=c+\delta S_{i}^{\prime }+\gamma_{i}$$

where:

*c* is the intercept;

δ represents the coefficients (to be estimated) associated with the characteristics of the individual *i*; and

$\gamma_{i}$ is the noise assumed to be normally distributed $N\left(0,\sigma_{\gamma }\right)$ .

The third component of the HCM framework is the measurement model component which relates the latent variable to its manifested indicators $I_{\mathrm{fi}}$ , for each individual *i*:

(3) $$I_{\mathrm{fi}}=d_{f}+\theta_{f}A_{i}+\mu_{\mathrm{fi}},\;\mathrm{with}\;f=1,\ldots ,F$$

where:

*f* is the number of equations associating the latent variable and the indicators;

$d_{f}$ is the intercept;

$\theta_{f}$ is the coefficient of the latent variable to be estimated; and

$\mu_{\mathrm{fi}}$ is the noise assumed to be normally distributed $N\left(0,\sigma_{\mu }\right)$ .

Following the normalization of Ben-Akiva et al., the first indicator, $d_{f}$ , has to be set equal to 0 while $\theta_{f}$ has to be set equal to 1 to guarantee the identification of the model (*36*).

Therefore, the probability of the individual i choosing a set of alternatives $j^{t}=\left(j^{1},\;\ldots \;,j^{T}\right)$ for the vector of choice tasks *T* can be written as the integral over the distribution of $\eta_{i}$ and $\gamma_{i}$ of the product of the conditional probability of choosing j in task *t*, $P_{\mathrm{jit}}\left(\eta_{\mathrm{ji}},\gamma_{i}\right)$ , the distribution of the latent variable, $g_{A}\left(\gamma_{i}\right)$ , and the product of the conditional distribution function of the indicators, $g_{I_{f}}\left(I_{\mathrm{fi}}|A_{i}\left(\gamma_{i}\right)\right)$ .

(4) $$P_{j^{t}i}\left(\eta_{ji},\gamma_{i}\right)=\int_{\eta ,\gamma }\prod_{t}P_{jit}\left(\eta_{ji},\gamma_{i}\right)g_{A}\left(\gamma_{i}\right)\prod_{f}g_{I_{f}}\left(I_{fi}|A_{i}\left(\gamma_{i}\right)\right)g\left(\eta \right)g\left(\gamma \right)d\eta d\gamma$$

The estimation of the joint HCM was performed through simulated maximum likelihood with the help of PythonBiogeme (*37*).

### Definition of Safety Factors

To identify the latent factors to test in the HCM, an exploratory factor analysis (EFA) was performed over the 14 psychometric statements (Table 3) associated with the perceptions of safety when the individual is traveling. First, indexes to evaluate the internal consistency and sample adequacy were calculated and all show very good figures. The Kaiser-Meyer-Olkin (KMO) test shows a very high level of sampling adequacy (KMO = 0.87) (*38*). Nonetheless, the value of the determinant of the Spearman correlation matrix (det = 0.0004 > 0.00001) indicates the absence of multicollinearity, while Bartlett’s test of sphericity (p-value < 0.01) confirms that the null hypothesis of having an identity matrix can be rejected (*39*, *40*). The EFA was performed through a principal axis factoring with varimax rotation, and the factor loadings are shown in Table 3. Choosing a cut-off of 0.61, large enough to retain the important statements and avoid overlap among different factors, the semantic exploration of the statements led to three latent factors characterizing the individual’s perception of safety while traveling: *worries of catching COVID-19; trust in safety measures*; and *dislike of quarantine*. Nonetheless, the Cronbach’s α of each identified latent factor is higher than 0.80 (i.e., 0.87, 0.87, 0.84, respectively). This shows high reliability in the indicators and, therefore, very good consistency in the Likert scale responses over the different questions (*41*).

**Table 3.** Factor Loadings from the Exploratory Factor Analysis (EFA).

Item	Statement	Worries of catching COVID-19	Trust in safety measures	Dislike of quarantine
**Item 1**	**I am afraid of catching COVID-19 for my health**	**0.68**	0.27	0.1
Item 2	I am afraid of passing COVID-19 to my family and friends	0.6	0.33	0.1
**Item 3**	**I would prefer not to travel to avoid catching COVID-19**	**0.75**	0.14	0.2
**Item 4**	**I would prefer not to travel to avoid quarantining**	0.29	0.14	**0.68**
**Item 5**	**I think it would be easy to catch the virus at the airport**	**0.73**	0.12	0.14
**Item 6**	**I think it would be easy to catch the virus on the airplane**	**0.74**	0.2	0.22
**Item 7**	**I will be safer wearing a mask at the airport**	0.23	**0.83**	0.06
**Item 8**	**I will be safer wearing a mask during my flight**	0.24	**0.89**	0.09
**Item 9**	**I will be safer having an empty seat between me and the next traveler**	0.31	**0.61**	0.21
**Item 10**	**I will not travel if I have to quarantine upon arrival**	0.09	0.18	**0.88**
**Item 11**	**I will not travel if I have to quarantine upon my return**	0.18	0.07	**0.75**
Item 12	I would not mind being tested at the airport	0.26	0.42	0.28
**Item 13**	**I am worried about meeting careless travelers during my flight**	**0.64**	0.38	0.25
Item 14	I would prefer for travelers to be prevented from carrying luggage in the cabin, in order to avoid contact with other passengers	0.49	0.26	0.11

*Note:* The items characterizing the three latent variables with a factor loading greater than 0.60 are highlighted in bold.

All the factors have been tested as latent variables in the HCM separately as well as jointly. However, only the first factor, *worries of catching COVID-19*, turned out to be statistically significant in the HCM.

## Results

The results of the estimation are presented in Table 4, showing two different model specifications: Model1 is a simple mixed model (ML) without the inclusion of latent variables, Model2 is the best specification of the estimated HCMs.

**Table 4.** Model Results

Name	Model1 (simple mixed model)		Model2 (hybrid choice model)	
	Value	t-test	Value	t-test
*Choice model component*				
Alternative specific constant (ASC)	4.12	6.80***	7.390	7.02***
*Fare (£100)*				
Long-haul, personal	−0.373	−11.18***	−0.371	−11.20***
Medium-haul, personal	−0.571	−10.87***	−0.569	−10.87***
Short-haul, personal	−1.01	−8.84***	−1.000	−8.85***
Long-haul, business	−0.249	−4.39***	−0.255	−4.66***
Medium-haul, business	−0.307	−3.82***	−0.315	−4.03***
Short-haul, business	−0.588	−3.20***	−0.604	−3.37***
*Time at the airport (h)*				
*Before flying out*				
Any distance, personal	−0.185	−4.94***	−0.183	−4.91***
Long/medium-haul, business	−0.181	−2.41**	−0.190	−2.62***
Short-haul, business	−0.283	−3.51***	−0.293	−3.75***
*After landing*				
Any distance, personal	−0.271	−5.91***	−0.268	−5.90***
Long-haul, business	−0.175	−2.56***	−0.181	−2.70***
Medium-haul, business	−0.268	−3.19***	−0.279	−3.40***
Short-haul, business	−0.313	−3.14***	−0.325	−3.37***
*Transfer*				
Long/medium-haul, personal	−0.127	−1.00	−0.123	−0.97
Short-haul, personal	−0.595	−4.39***	−0.590	−4.36***
Any distance, business	−0.392	−2.18**	−0.410	−2.34**
*Sociodemographic*				
Age: 45 years old or above	−1.43	−3.87***	−1.340	−3.44***
Full-time employment	1.24	3.32***	1.080	2.76***
Income: £50k p.a. or above	−2.06	−2.86***	−2.500	−3.36***
*Video calls and virtual activities*				
Used during COVID-19 outbreak, business	3.69	3.32***	3.870	3.56***
Will be used after COVID-19 outbreak, business	−2.84	−2.66***	−3.130	−2.95***
Error component	−2.92	−13.35***	2.830	13.39***
*Latent variables*				
Worries of catching COVID-19			−0.882	−3.81***
*Structural model component*				
Below 45 years old			−0.246	−2.79***
Female			0.335	3.72***
Big Five: Agreeableness (skeptical)			0.179	2.03**
LV Constant			3.630	43.32***
LV γ			−0.263	−3.49***
*Measurement model component*				
Intercept indicator 2 (Item 3)			−0.650	−2.17**
Intercept indicator 3 (Item 5)			0.262	0.83
Intercept indicator 4 (Item 6)			−0.160	−0.43
Intercept indicator 5 (Item 13)			0.548	1.74
Coefficient indicator 2 (Item 3)			1.190	15.44***
Coefficient indicator 3 (Item 5)			0.934	11.45***
Coefficient indicator 4 (Item 6)			1.110	11.59***
Coefficient indicator 5 (Item 13)			0.975	12.01***
Standard deviation indicator 1 (Item 1)			−0.187	−3.51***
Standard deviation indicator 2 (Item 3)			−0.324	−5.53***
Standard deviation indicator 3 (Item 5)			−0.501	−8.83***
Standard deviation indicator 4 (Item 6)			−0.540	−7.84***
Standard deviation indicator 5 (Item 13)			−0.395	−6.68***
*Number of draws*		500		500
*Number of parameters*		23		42
*Sample size*		2,316		2,316
*Initial log-likelihood*		−2,544.39		−6,328.32
*Final log-likelihood*		−1,863.43		−4,260.12
*Rho2*		0.268		0.327
*Adjusted Rho2*		0.259		0.32

*** p-value ≤ 0.01.

** 0.01 < p-value ≤ 0.05.

### Model Specification Search

The model is characterized by the utility functions of the three alternatives: two travel alternatives and a no-travel alternative. The two travel alternatives are defined by the attributes obtained through the SP design, as described previously. The no-travel alternative is the reference case with the utility set to zero (i.e., no attributes define this alternative). Different model specifications were tested during the estimations to obtain significant and robust model results. All the attributes of the SP design and the error component to consider the serial correlation across SP choices were included in the models. The socio-economic characteristics available were all tested as categorical variables drawing on the different ranges illustrated in Table 1.

Nonetheless, the travel purpose deserves a more accurate explanation. Indeed, the respondents were re-contacted for a second wave of interviews and, during the SP, were asked to think about a hypothetical air travel trip for the same purpose as the trip analyzed during the first wave of interviews. Therefore, this variable was used to segment the attributes of the alternatives during the estimations. The other segmentation that needed to be taken into account to capture preference heterogeneity was the type of flight (i.e., short-haul, medium-haul, or long-haul) as the attribute levels varied by type of flight.

Concerning the COVID-19-related questions, the statements to explore the safety concerns of the respondents when traveling were used as indicators of the latent variables. The questions concerning the frequency of video calls with family and friends living in other cities (in the U.K. or abroad) and the use of online/virtual software in place of flying for business/work before the pandemic and during the pandemic were treated as dummy variables (equal to 1 for the frequency of several times a month or more, and 0 otherwise), while their anticipated use after the pandemic was treated as 1 when the anticipated frequency was “more” or “much more” than before the COVID-19 outbreak, 0 otherwise. The questions to investigate Big Five personality traits were used to generate a set of dummy variables which take the value 1 when the person agreed or strongly agreed with the personality trait, 0 otherwise.

### Choice Model Component

All the coefficients of the attributes characterizing the two travel options were kept generic in the models presented in Table 4. Making the coefficients alternative-specific did not improve the model fit and, in this specific context where individuals had to choose between two unlabeled alternatives, would not add any particular value in explaining the decision-making process. Instead, the coefficients of the design attributes were segmented for the type of flight (short-, medium-, long-haul) and purpose of the trip (personal/business). The only variable that required a different segmentation to obtain more robust and statistically significant results is the coefficient on the transfers. It was kept generic across the type of flight for business trips as well as across the type of flight medium-haul and long-haul for personal trips.

Overall, in both models, the coefficients on the attributes of the alternatives are all significant at above the 95% confidence level with the expected (negative) sign, because of the extra burden on the traveler. The only variable which resulted in an insignificant parameter is the presence of transfers for medium- and long-haul personal trips, suggesting that people do not care about transfers (or are used to transfers) when traveling medium or long distances for personal reasons.

Considering the fare per flight type, as expected, it is observed that the sensitivity to cost for personal travel is always higher than the sensitivity to cost for business travel. Moreover, this sensitivity decreases when the haul distance increases. Concerning the time taken at the airport, it is also clear that travelers are more sensitive to the time spent at the arrival airport than the time spent at the departure airport. For business trips, the sensitivity decreases when the haul distance increases, possibly reflecting their experience of longer time spent at airports for long-distance travel (e.g., because of passport checks, waiting time for the luggage) while the distance segmentation is not statistically different for personal trips.

Nonetheless, there is generally a lower willingness to travel in the pandemic scenario among people above 45 years and among people with a household annual income of £50k–£100k or more. These results could reflect some combination of a degree of anxiety that COVID-19 is reported to be more dangerous for older people, and the ability of older and more well-established individuals to avoid risky situations and avoid traveling (either because they are in a more senior position at work or because they have fewer familial responsibilities that necessitate travel). On the contrary, the model results suggest that full-time employed individuals are more likely to travel, possibly because of a combination of their work-related duties (to maintain their employment or business) and being covered by sick leave provisions.

The impact of the frequency of video calls on air travel choices is only significant for the business travel segment. Specifically, it appears that having frequently used online/virtual software in place of flying for business/work *during* the pandemic has generated the need or the will to travel again when it will be possible. However, having frequently used online/virtual software in place of flying for business/work *before* the pandemic does not have a significant effect on the choices. This could indicate the presence of a segment of business travelers for whom the video calls are a strictly temporary measure. Alternatively, it is also possible that the recent experience of extensive video calling has created a saturation effect that increases the desire to travel, while “past states” (i.e., whether video calls were used before the pandemic) are too far away to matter. On the other hand, as expected, the probability of traveling decreases for people who think that *after* the pandemic online/virtual software will be used more or much more than before the COVID-19 outbreak.

There is also a general preference to travel (compared with the no-travel option) as indicated by the positive alternative specific constant (ASC) generically estimated across the two travel options. Error components were included in the generic form for the two travel options to consider the individual-specific panel effect that potentially generates serial correlation across the SP choice situations for the same respondent. As anticipated, the parameter of the error component is statistically significant. Therefore, the two traveling options are characterized by a substantial amount of unobserved preference heterogeneity. This unobserved preference heterogeneity might result from unobserved attitudes and is partially captured in Model2 by the latent variable, also included in the utility of the travel options, which explains the different values of the error component parameters between Model1 and Model2 (*42*).

The only latent variable that came up statistically significant to explain the heterogeneity of the air travel choice with respect to COVID-19 is “Worries of catching COVID-19”. In particular, this variable was included with a generic parameter in the two traveling options. In line with intuition, it was estimated to be negative and highly statistically significant, which indicates that people who worry about catching COVID-19 at the airports or on-board the airplane, or worry about meeting careless travelers, are less likely to travel under the current (pandemic) conditions. As it is possible to see in Table 4, the inclusion of the latent variable “Worries of catching COVID-19” did not interfere with the magnitude nor the significance of the variables already present in Model1 (i.e., the simple ML), showing the consistency and robustness of the model results.

As discussed, no significant role is observed for the variable “trust in safety measures” (i.e., use of masks and empty seats) or the variable “dislike of quarantine”. This may be related to several factors such as the stage of the pandemic during which the survey was conducted (the first lockdown in the U.K. was eased in June and the SP wave of data collection occurred in July, consequently there was likely to be a greater sense of optimism), the rather inconsistent official recommendations on masks issued in the U.K. at that time, and the toughness (or lack therefore) of the quarantine rules in the U.K. However, additional information is required to better investigate this matter.

### Structural Model Component

The structural model component illustrates the characteristics of the people with an underlying latent concern of catching COVID-19. Two main pieces of information were used for this purpose: the socio-economic characteristics and the Big Five personality traits of the respondent. The model estimation suggests that the latent concern of catching COVID-19 is positively correlated with people that are female, and negatively correlated with people younger than 44 years. This is consistent with the negative estimate in the choice model component of the “older than 45 years” variable, which suggests that there is a lower inherent preference toward traveling of this demographic group that is at a higher risk of having severe consequences from COVID-19 (*43*).

With regards to personality traits, this latent construct in the structural model component is positively and significantly correlated with a reverse indication of “agreeableness”, that is, the propensity to see oneself as someone who tends to find fault with others. This suggests that individuals who are “not agreeable”, that is, are generally suspicious of others, are likelier to have a latent concern of catching COVID-19.

### Measurement Model Component

In the measurement model component, all the five coefficients manifesting the latent variable are significant at the 95% confidence level or above. This confirms the results of the exploratory factor analysis and the presence of correlation among the indicators. Also, the results show that the indicators are accurately manifesting the latent variable construct. In other words, the latent variable “Worries of catching COVID-19” includes people who are afraid of catching the virus because of its potential impact on their health, think it would be easy to catch the virus at the airport or on-board the airplane, and are worried about meeting careless travelers during their flight.

Looking at the outcomes of the structural and measurement model components to help with the recovery of the air travel sector in the near future, actions and campaigns targeting the segment of the population that is more concerned would be important to help recapture the demand. In particular, a better and more consistent explanation, and its dissemination, of how airports and airlines guarantee the safety of the passengers and work to minimize the risk of infections is needed. Besides, market research efforts ought to be directed at this segment to investigate ways by which suitable assurance could be generated for this group.

### Trade-Off Analysis

According to the estimation results of Model2, the segmented trade-off values for the design attributes (besides “transfers, personal trips, long/medium-haul” which were not statistically significant) are presented in Table 5. As suggested by the sensitivity of the estimates, the WTP of business travelers is, in general, higher than that of travelers for personal trips, and increases with the distance traveled, which is consistent with previous literature (*18*–*20*). Moreover, it is noted that the value of time spent at the departure airport is on average £33 per hour for personal flights and £61 per hour for business flights, and the value of time spent at the arrival airport is on average £49 per hour for personal flights and £71 per hour for business flights. The higher WTP to reduce the time spent at the arrival airport compared with the departure airport is arguably quite intuitive.

**Table 5.** Segmented Trade-Off Values

	Willingness-to-pay (WTP)
Time at the airport:	WTP for -1h (£/h)
*Before flying out*	
Long-haul, personal	49
Medium-haul, personal	32
Short-haul, personal	18
Long-haul, business	75
Medium-haul, business	60
Short-haul, business	49
*After landing*	
Long-haul, personal	72
Medium-haul, personal	47
Short-haul, personal	27
Long-haul, business	71
Medium-haul, business	89
Short-haul, business	54
Transfer:	WTP for non-stop (£)
Short-haul, personal	59
Long-haul, business	161
Medium-haul, business	130
Short-haul, business	68

The values of time at the departure and arrival airports estimated in this study are not directly comparable with other studies as these attributes become particularly relevant in the presence of possible COVID-19 safety measures (e.g., time needed for a test before the departure or after arrival). However, these figures are similar to the WTP for a reduction in access time to airports that are observed in previous studies, such as on average $68 per hour in Warburg et al. (*19*). Finally, the WTP for non-stop flights found in this paper (on average £104) seems higher than the values reported by Warburg et al., who estimated $69 on average, and by Hess et al., who estimated $44 for business and $20 to $62 for holiday (*19*, *20*). This discrepancy is likely related to the fact that, in pandemic conditions, a transfer is associated with potentially more social contact and a correspondingly higher risk of contracting the virus.

## Conclusions

The COVID-19 pandemic and associated travel restrictions have created an unprecedented challenge for the air travel industry. The slump in demand and the continuing uncertainty about medium- and long-term impacts on people’s willingness to travel by air warrant studies that shed light on people’s attitudes toward traveling by air during and after the pandemic. It is hoped that such insights can aid the management and the recovery of the sector during these particularly difficult times.

To that end, the current study presents two novel contributions to the discipline. First, to the best of the authors’ knowledge, this is the first SP survey design for air travel developed to explicitly account for disruptions caused by COVID-19, which also reflects changes in the trade-off variables such as a possible increase in times and costs at airports. Second, this is the first survey-based analysis that quantifies how safety perceptions and attitudes may interact with the traditional time-cost trade-offs made by air passengers in selecting a travel itinerary with pandemic-driven uncertainty.

The results of this U.K. case study confirm the impact of people’s worries of catching COVID-19 on their willingness to travel, providing further sociodemographic segmentation and quantifying those effects against other considerations, including the cost of travel or number of transfers. At the same time, no effect was observed with respect to perceptions of safety because of wearing a mask or being concerned about the necessity to quarantine. It is noted that this is potentially a U.K.-specific finding because of the inconsistent official recommendations on masks issued in the U.K. at that time, and the lack of enforcement of quarantine rules in the U.K. at the time of data collection. In addition, the results suggest that some respondents, especially business travelers, might be perceiving virtual (video calling) substitutes of air travel to be only a temporary measure while seeking to return to traveling as soon as possible. This may well be a piece of positive outlook for the air travel industry, since business travelers, before the pandemic, were twice as profitable as the rest of the travelers, which means they accounted for as much as 75% of the profits of the airlines (*44*, *45*).

This study is an ongoing piece of research, and the research is currently being expanded to undertake a comparative analysis that will include air passengers from New York, Shanghai, and Sao Paulo, in addition to London. The RP and SP data from each of these cities is also being explored to uncover additional, arguably more sophisticated, relationships between preferences and attitudes toward traveling by air, especially considering that the data from the three other cities have been collected at slightly different points in time and under different public health conditions.
